# Spatial patterns of lower respiratory tract infections and their association with fine particulate matter

**DOI:** 10.1038/s41598-021-84435-y

**Published:** 2021-03-01

**Authors:** Aji Kusumaning Asri, Wen-Chi Pan, Hsiao-Yun Lee, Huey-Jen Su, Chih-Da Wu, John D. Spengler

**Affiliations:** 1 Department of Geomatics, National Cheng Kung University, Tainan, 70101 Taiwan; 2 Institute of Environmental and Occupational Health Sciences, National Yang Ming Chiao Tung University, Taipei, 11221 Taiwan; 3 Department of Leisure Industry and Health Promotion, National Taipei University of Nursing and Health Sciences, Taipei, 112 Taiwan; 4 Department of Environmental and Occupational Health, National Cheng Kung University, Tainan, 70101 Taiwan; 5 National Institute of Environmental Health Sciences, National Health Research Institutes, Miaoli, 35053 Taiwan; 6 Department of Environmental Health, Harvard T.H. Chan School of Public Health, Boston, 02115 USA

**Keywords:** Diseases, Infectious diseases, Respiratory tract diseases

## Abstract

This study aimed to identify the spatial patterns of lower respiratory tract infections (LRIs) and their association with fine particulate matter (PM_2.5_). The disability-adjusted life year (DALY) database was used to represent the burden each country experiences as a result of LRIs. PM_2.5_ data obtained from the Atmosphere Composition Analysis Group was assessed as the source for main exposure. Global Moran’s I and Getis-Ord Gi* were applied to identify the spatial patterns and for hotspots analysis of LRIs. A generalized linear mixed model was coupled with a sensitivity test after controlling for covariates to estimate the association between LRIs and PM_2.5_. Subgroup analyses were performed to determine whether LRIs and PM_2.5_ are correlated for various ages and geographic regions. A significant spatial auto-correlated pattern was identified for global LRIs with Moran’s Index 0.79, and the hotspots of LRIs were clustered in 35 African and 4 Eastern Mediterranean countries. A consistent significant positive association between LRIs and PM_2.5_ with a coefficient of 0.21 (95% CI 0.06–0.36) was identified. Furthermore, subgroup analysis revealed a significant effect of PM_2.5_ on LRI for children (0–14 years) and the elderly (≥ 70 years), and this effect was confirmed to be significant in all regions except for those comprised of Eastern Mediterranean countries.

## Introduction

Lower respiratory tract infections (LRIs), such as pneumonia and bronchiolitis, have been a public health concern for decades due to the severity of illnesses^1,2^. A global study in 2016 has reported that LRIs are the sixth-leading cause of death for all ages and the leading cause of death in children younger than 5 years of age^2^. In total, LRI resulted in 2,377,697 deaths (95% Uncertainty Interval [UI]: 2,145,584–2,512,809), which includes a high number of fatalities in children younger than 5 years old (652,572 deaths, 95% UI: 586,475–720,612) and in elderly people who are at least 70 years old (1,080,958 deaths; 95% UI: 943,749–1,170,638). Previous studies have confirmed the determinants of LRIs include age, parental and caregiver status, comorbidities (e.g., measles, diarrhea, malaria), and environmental factors^3^. A report released by the United Nations International Children’s Emergency Fund in 2016 indicated nearly 50% of deaths caused by LRIs occur in sub-Saharan Africa^4^. In connection with this issue, several studies related to LRIs especially for children younger than 5 years old have been conducted^5–7^. In addition, a 2016 Global Burden of Disease study asserted emphasizing geographic disparities is the key to reducing fatal outcomes worldwide of LRIs^8^. Therefore, to reduce prevalence of LRIs, more attention should be directed to spatial studies rather than concentrating intervention efforts at the individual level.

In order to correctly identify critical areas of LRIs, a more precise analytic method needs to be applied. Spatial statistical analysis within spatial epidemiology has become indispensable in guiding targeted interventions. Spatial epidemiology studies reach beyond the purview of general spatial statistics by investigating geographic health data with respect to demographic, behavioral, environmental, socioeconomic, and other risk factors^9^. Previous studies have applied spatial analysis to determine health outcome hotspots and have confirmed that these approaches are widely used in epidemiology studies to identify spatial patterns and hotspots of infectious diseases^10–12^. In regards to the spatial pattern of LRIs, prior studies conducted in Ethiopia yielded limited research findings because of their reliance on traditional spatial statistics^3,13,14^. In contrast, one study applied Global Moran’s I and SaTScan and discovered the acute respiratory infection spatial pattern among children younger than 5 years old in Ethiopia (Moran’s I = 0.34)^15^. Moreover, LRI clusters were found in the Tigray and Oromia regions^15^. Across various studies, utilization of spatial statistical approaches, such as dot maps, rate maps, Moran’s I, and Getis-Ord Gi*, in studying LRIs and other diseases have provided better performance as compared to traditional methods^16–19^.

In addition to the relationship between LRIs and spatial factors, previous studies have noted a strong relationship between LRIs and air pollutants. Beamer applied multivariable regression analyses to investigate the effects of air pollution on health and demonstrated LRIs for which wheezing is a symptom were associated with increased air pollution and, specifically, the presence of 25 different chemical components (OR 1.18, 95% confidence interval [CI] 1.04–1.35)^20^. Among the litany of air pollutants, fine particulate matter (PM_2.5_) has been confirmed to be associated with premature death^21–23^. According to the 2010 Global Burden of Disease study, PM_2.5_ was ranked as one of leading risk factors, contributing to 3.1 million deaths and accounting for 3.1% of global disability-adjusted life years (DALYs)^24–26^. Moreover, it was estimated for 2015 that 4.2 million (95% CI 3.7–4.8 million) deaths and 103.1 million (90.8–115.1 million) DALYs were associated with PM_2.5_^27^. Fortunately, the negative impacts of air pollutants can be reduced by greenness, leading to a decrease in disease burden and mortality^28–32^. Although the diametric impacts of air pollutants and greenness on health are known, few studies have investigated their relationship with LRIs. Accordingly, in addition to spatial determinants, this study includes air pollutant levels and greenness in its analysis.

To our knowledge, only a few studies have investigated the relationship between LRIs and PM_2.5_ on a global scale and even fewer have considered levels of greenness exposure. Therefore, this study aims to utilize sophisticated analyses in order to examine the association on a global scale between LRIs and their determinants. By using a country-level database of 183 countries worldwide, this ecological study sought to identify the spatial pattern distribution of LRIs in determining the most critical regions and investigate its linkage with PM_2.5_ exposure. Global Moran’s I and Getis Ord-Gi* were applied for spatial pattern analysis. A generalized linear mixed model coupled with sensitivity tests and subgroup analysis were then estimated to clarify associations with PM_2.5_ in various circumstances. Since previous studies have confirmed that PM_2.5_ can increase the burden of LRIs, we assumed PM_2.5_ would be positive linked to LRIs globally.

## Results

### Descriptive statistics

Descriptive statistics for country-level variables at the baseline (data in 2000) are displayed in Table 1. The mean population density was estimated at 132 people per km^2^. Moreover, nearly half of the population was male and fell within the age range of 15–49 years. For health behaviors, the annual alcohol consumption and smoking prevalence were 4.68 l/population and 22.14%, respectively. Lastly, the average percentage of healthcare expenditure was 6.61% of Gross Domestic Product or GDP (SD = 2.74% of GDP). Regarding exposures, the mean PM_2.5_ concentration was 17.62 µg/m^3^ (SD = 14.44 µg/m^3^), the mean temperature was 19.29 °C (SD = 8.00 °C), and the average wind speed was 6.37 m/s (SD = 1.48 m/s). At the baseline, the mean disability-adjusted life year (DALY) due to LRIs was 41.21 years (SD = 41.99 years) per 1000 population. This indicates that among 1000 people, we expect to observe 41.21 years lost to illness or premature death due to LRI. The trends of DALY due to LRIs are displayed in Supplementary Fig. S4 and they reflect that Africa had the highest burden among all of the regions, though its DALY continually decreased over the study periods.

**Table 1:** Baseline characteristics of variables.

Variable	Mean	Std. dev	Min	25th	Median	75th	Max
**Covariates**							
Population density (people/km^2^)	132.78	561.10	0.49	17.16	45.38	89.46	7249.61
Gender (male %)	49.87	2.30	45.96	49.05	49.60	50.20	67.49
Age 0–4 (years, %)	11.46	4.68	4.03	6.97	11.23	15.15	20.99
Age 5–14 (years, %)	21.47	5.89	9.72	15.84	22.74	26.65	31.28
Age 15–49 (years, %)	50.27	4.30	40.37	47.28	49.97	53.15	67.92
Age 50–69 (years, %)	12.40	5.59	5.51	8.06	9.88	16.70	26.67
Age > 70 (years, %)	4.40	3.30	0.60	1.84	2.90	6.71	13.02
Economic—income level (categorical variable)	–	–	–	–	–	–	–
Alcohol consumption (liters/population/year)	4.68	4.06	0.00	12.23	3.78	7.57	14.06
Smoking (%)	22.14	16.26	0.00	8.25	22.70	34.35	73.40
Healthcare expenditure (% of GDP)	6.61	2.74	0.00	4.80	6.35	8.39	17.14
Temperature (°C)	19.29	8.00	− 6.21	11.80	22.78	25.71	28.74
Wind speed (m/s)	6.37	1.48	3.09	5.27	6.41	7.41	10.41
**Exposures**							
PM_2.5_ (µg/m^3^)^a^	17.62	14.44	0.46	7.42	14.67	23.14	67.13
NDVI (normalized difference vegetation index)^b^	0.47	0.20	0.08	0.33	0.51	0.63	0.82
**Health outcome**							
DALY loss due to LRIs (years)	41.21	41.99	1.68	6.68	14.85	63.11	235.03

^a^Main exposure.

^b^Exposure for subgroup analysis.

### Spatial pattern and hotspot analysis

Moran’s I was utilized in spatial statistics analyses to determine whether or not LRIs are clustered in certain areas. Table 2 lists the results of Moran’s I and reflects the spatial autocorrelation of DALY loss due to LRIs during the study periods (i.e., 2000, 2010, 2015, 2016). The positive value of Moran’s index means countries have DALY values that are similar to those of the countries surrounding them. The results reveal the value of Moran’s I was 0.79 (z-score = 12.70; *p* < 0.001), which confirms that a clustered pattern was observed. This result is consistent with visual inspections of hotspot analyses.

**Table 2:** Global Moran’s I summary of DALY loss due to LRI for each period analyzed.

Year	Moran’s Index	z-score	p-value	Spatial distribution
2000—2016	0.792	12.699	< 0.001	Clustered
2000	0.767	12.275	< 0.001	Clustered
2010	0.770	12.392	< 0.001	Clustered
2015	0.748	12.081	< 0.001	Clustered
2016	0.747	12.070	< 0.001	Clustered

**Figure 1:**
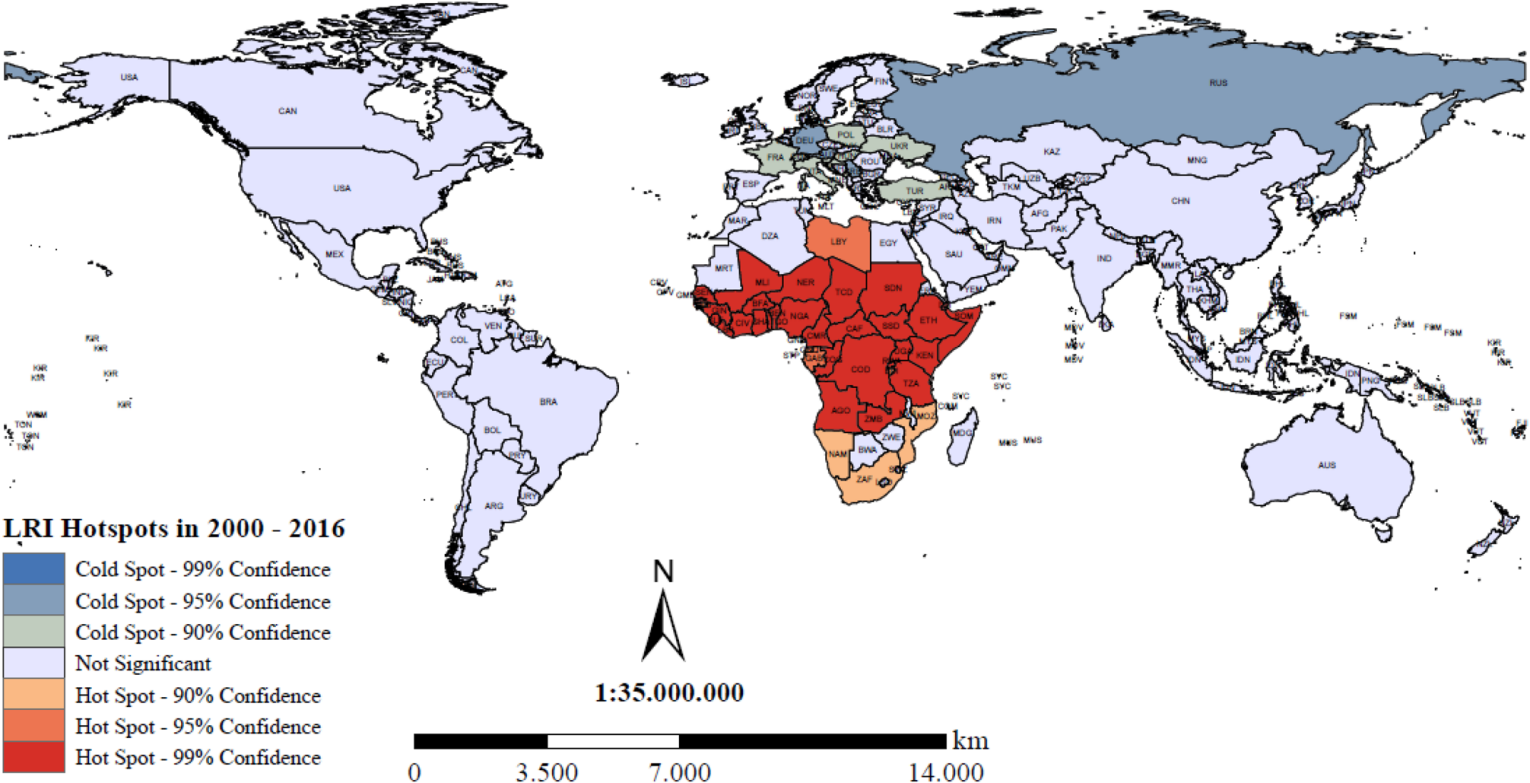
Spatial hot spots and cold spots of DALYs due to LRI.

With regard to hotspot analysis, the location of clustering was determined using Getis-Ord Gi*. The hot spot areas were marked red while the cold spot areas were marked blue. As illustrated in Fig. 1, 35 countries in Africa and 4 countries in the Eastern Mediterranean were marked as hotspot areas, which suggests these countries have the highest burden of LRIs as compared to all of the other countries. In contrast, the cold spot areas are dominated by 18 low-DALY countries in Europe. We further examined changes in area, values, and confidence intervals of hot spots due to LRIs by years. These results are displayed in Supplementary Fig. S6a–d. In addition, we used the row-standardized weights matrix for the global measures given earlier to investigate the statistical significance of z-scores (Gi*) assigned to each country, and this revealed the presence and intensity of local clusters of hot spots and cold spots. Confidence intervals of 90%, 95%, 99% were reported for each of these in Supplementary Fig. S7a–e.

### Association model and sensitivity test

Table 3 lists the results of the statistical association models measuring the significance of the association between PM_2.5_ and LRIs. After adjusting for pertinent covariates (demographics, socioeconomic status, healthcare status, lifestyle behaviors, and meteorological factors), the main model yielded a significant positive correlation between exposure to PM_2.5_ and LRIs, with a coefficient of 0.21 (95% CI 0.06–0.36; *p* < 0.01) in changes in DALY based on an increase of 1 µg/m^3^ PM_2.5_. This result indicates PM_2.5_ may increase the disease burden of lower respiratory infections globally. The results of five sensitivity tests with different covariate adjustments (Model 1 to Model 5) and an additional test that eliminated Eastern Mediterranean countries (Model 6) again confirm the positive association between PM_2.5_ and LRIs. Model 1 controlled for demographic factors and yielded a PM_2.5_ coefficient of 0.20 (95% CI 0.05–0.36). Model 2 controlled for demographics and alcohol consumption and its PM_2.5_ coefficient was estimated as 0.21 (95% CI 0.05–0.36). Model 3 controlled for demographics and alcohol consumption as did model 2, however it further controlled for smoking, and its PM_2.5_ coefficient was 0.21 (95% CI 0.06–0.37). Model 4 built upon model 3 and included economic status additionally, and its PM_2.5_ coefficient was 0.21 (95% CI 0.06–0.36). In model 5, healthcare expenditure was included in addition to all of model 4’s covariates, and its PM_2.5_ coefficient was 0.20 (95% CI 0.05–0.35). Lastly, model 6 excluded Eastern Mediterranean countries, which yielded a significant and positive PM_2.5_ coefficient of 0.42 (95% CI 0.21–0.63).

**Table 3:** Association models for LRI and PM_2.5_, controlled for various covariates.

Model	Coefficient of PM_2.5_ (95% confidence interval [CI])	p-value
Main model^a^	0.207 (0.058–0.356)	< 0.01
**Sensitivity test adjusted for covariates**		
Model 1^b^	0.204 (0.051–0.357)	< 0.01
Model 2^c^	0.205 (0.052–0.358)	< 0.01
Model 3^d^	0.212 (0.059–0.365)	< 0.01
Model 4^e^	0.211 (0.062–0.362)	< 0.01
Model 5^f^	0.200 (0.051–0.347)	< 0.01
Model 6^g^	0.420 (0.212–0.627)	< 0.001

^a^Control variables included density of population, sex (% of male), age, smoking, alcohol consumption, economic status, healthcare expenditure, temperature, and wind speed.

^b^Adjusted for density of population, sex (% of male), and age.

^c^Adjusted for density of population, sex (% of male), age, and alcohol consumption.

^d^Adjusted for density of population, sex (% of male), age, alcohol consumption, and smoking.

^e^Adjusted for density of population, sex (% of male), age, alcohol consumption, smoking, and economic status.

^f^Adjusted for density of population, sex (% of male), age, alcohol consumption, smoking, economic status, and healthcare expenditure.

^g^Considered all covariates and eliminated data from Eastern Mediterranean countries.

### Subgroup analysis

**Figure 2:**
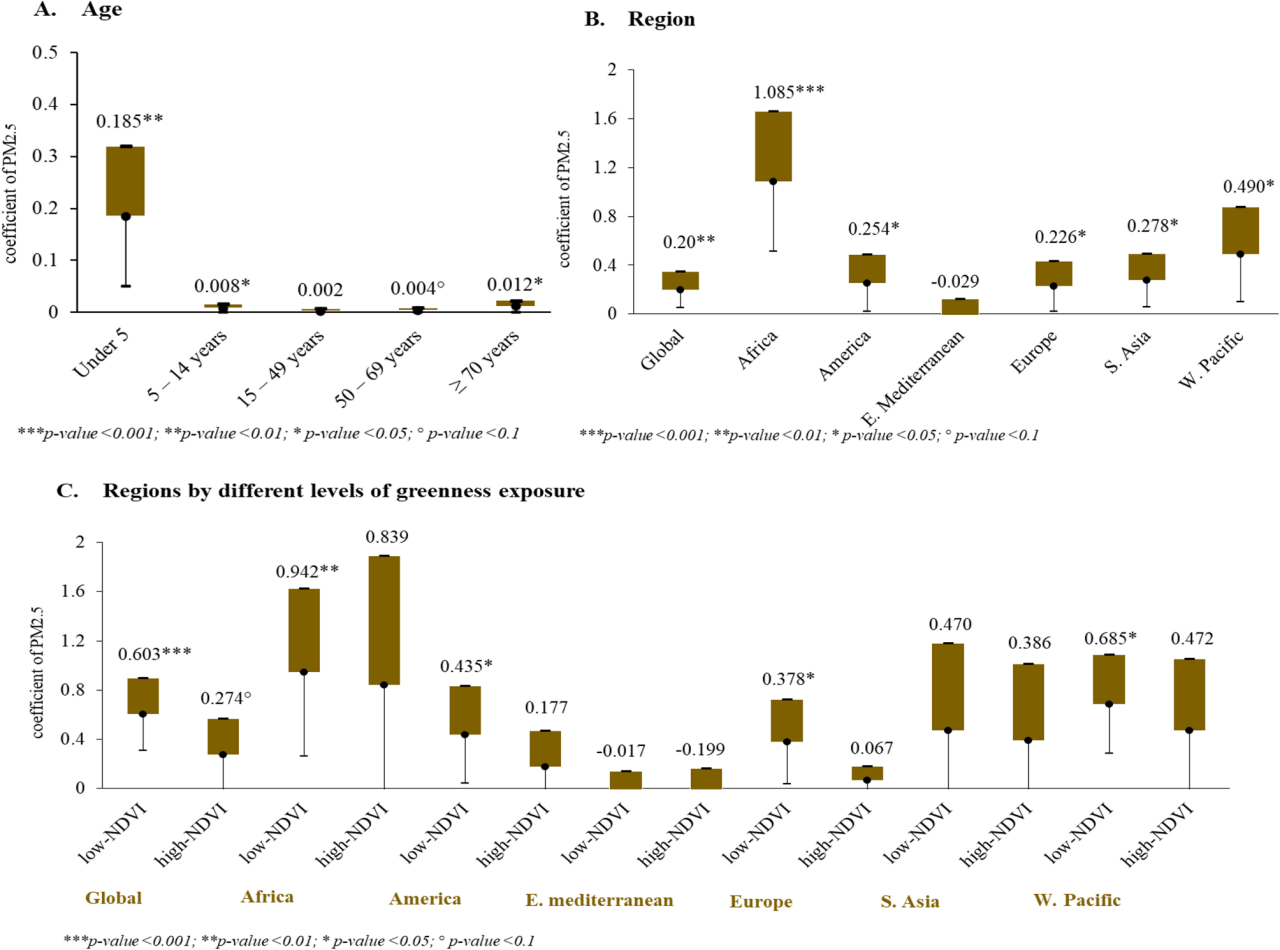
Subgroup analysis, stratified by (**A**) age, (**B**) WHO region; and (**C**) WHO Regions by different levels of greenness.

Figure 2 illustrates the respective association levels between exposure to PM_2.5_ and LRIs for various subgroups, for instance, by age group, by WHO region, and by WHO regions according to level of greenness exposure. Regarding age groups, the results reflect a positive association between PM_2.5_ exposure and LRIs in children (0–14 years old) and the elderly (≥ 70 years old). Moreover, there was marginal significance in mature adulthood (50–69 years old). Among all age groups, children younger than 5 years old had the highest positive value for association (coefficient = 0.19; 95% CI 0.05–0.32). When conducting subgroup analysis of the six WHO regions, a significant positive association between PM_2.5_ and LRI was found for each region except for the Eastern Mediterranean. The overall findings strengthen scientific evidence that the adverse effects of PM_2.5_ exposure can increase the burden of LRIs in nearly all regions around the world. It is noted the African region had the highest value of PM_2.5_ (coefficient = 1.09; 95% CI 0.51–1.66), which is consistent with the results of the spatial hotspot analysis (see Fig. 1). Lastly, a subgroup analysis was performed for WHO regions according to their level of greenness. The global results revealed that the effect of PM_2.5_ was significantly higher in low greenness countries (coefficient = 0.60; 95% CI 0.31–0.90), while countries with high NDVI had marginally significant results. When analyzing results by regions, significant positive associations between PM_2.5_ and LRIs were found in low NDVI countries in the African, American, European, and Western Pacific regions, where the coefficients were 0.94, 0.44, 0.38, and 0.69, respectively. In contrast, low NDVI countries in Southeast Asia and the Eastern Mediterranean did not display any significance.

## Discussion

Although the associations between global disease burden and mortality from lower respiratory infections and particulate matter have been studied^33–36^, few studies have investigated the spatial pattern of LRIs and analyzed its association with PM_2.5_ and then linked it to greenness exposure levels. In order to provide a complete and detailed picture of LRIs, this study applied sophisticated analytic methods to examine the spatial pattern and hot spots of LRIs, as well as their association with PM_2.5_, then linked them to greenness exposure levels at the global scale in analysis. Findings of this study indicate Africa is the region with the highest burden due to LRIs, although its burden trend has gradually been declining. Our findings are consistent with prior studies in which LRIs were estimated to account for 38.6% of death by infectious disease and 14.9% of overall deaths in African children^37^. For these reasons, Africa has become a top priority in addressing infectious diseases, especially LRIs^38^. It should be noted the identification of hotspot areas not only identifies areas with the highest rate of disease but also reveals how spatial proximity plays a role in the spread of disease in Africa. In contrast, cold spot areas of LRIs were detected in Europe, suggesting this region has the lowest burden resulting from LRIs. This finding is reinforced by Nowbar’s finding that cardiovascular diseases are more common in Europe^39^.

The global model found a significant positive association between PM_2.5_ and LRIs, even after controlling for covariates. Consistent significant positive associations were also yielded in the ensuing sensitivity tests, and this indicates that a higher level of exposure to country-level PM_2.5_ is associated with a higher disease burden due to LRIs. Further, in our sensitivity analysis, model 6 indicated a large increase in the estimate of the PM_2.5_ coefficient compared to the other five sensitivity models. In this case, a plausible explanation is that in this model we omitted data for Eastern Mediterranean countries that have high PM_2.5_ values, where these values could possibly exist as outliers. Thus, by removing outliers in the model calculation (model 6), an increase in the coefficient estimation and/or correlation significance was possible. In general, our study findings are supported by prior studies; for example, Liu’s study demonstrated PM_2.5_ exposure can increase the risk of experiencing pneumonia and bronchitis, the two most common lower respiratory infections^40^. Moreover, a study conducted in the United States reported that PM_2.5_ has a significant effect on the severity of health outcomes for people experiencing LRIs^41^. Plausible reasons for this, such as how ambient air pollutants, including PM_2.5,_ deteriorate respiratory health, have been discussed in other studies^42–44^. PM_2.5_ impairs the defense function of airway epithelial hosts by altering respiratory microecology and inducing immune cell dysfunction^45^. Further, in Yang et al.’s study, they addressed how PM_2.5_ can damage the capacity of the immune system, particularly in the respiratory tract, which results in humans being more vulnerable to infection^45^.

According to the age-stratified analysis, a positive association exists between PM_2.5_ and LRIs across many age groups. Moreover, statistical significance was present for children (i.e., < 5 years old and 5–14 years old) and the elderly (≥ 70 years old). The effects of PM_2.5_ in damaging the respiratory system of children, particularly those younger than 5 years old, were supported in Egondi’s study^46^. He concluded that high levels of exposure to outdoor PM_2.5_ is linked to a high child mortality rate resulting from respiratory problems, with an incidence rate ratio of 1.12 (95% CI)^46^. Another study, this one conducted in China, revealed a significant association between PM_2.5_ and hospital visits for acute upper and lower respiratory infections among children younger than fifteen years old^47^. With a focus on children in low-middle income countries, Lelieveld et al. concluded that ambient PM_2.5_ accounted for approximately 5% of deaths due to LRIs and accounted for 18% of losses in life expectancy^48^. Compared to adults, children are much more vulnerable to air pollutants because they inhale a higher air volume per body weight than do adults and their immune systems are not yet mature^49^. In addition to its impact on children, PM_2.5_ also had an impact on the elderly, and numerous studies have provided evidence supporting the elderly to be a vulnerable group^50–52^.

We further investigated the association between PM_2.5_ exposure and LRIs in all six WHO regions. A significant positive association was found in each region (i.e., Africa, America, Europe, Southeast Asia, and the Western Pacific) except for the Eastern Mediterranean. A retrospective study conducted in Cameroon concluded that dry and dusty weather is a source of PM_2.5_ and increases the risk of acute respiratory infections in Africa^53^. The WHO Regional Office for Africa reported that more than 50% of premature deaths due to pneumonia among children younger than 5 years old are caused by the particulate matter^54^. PM_2.5_ concentrations being associated with an increase in the incidence of acute lower respiratory infections has also been confirmed by several observational studies in Southeast Asia^55,56^, America^57,58^, Europe^59,60^, and the Western Pacific^44,61–63^. In a collaborative study, Burnett reported global estimates of specific mortality in terms of non-communicable diseases and lower respiratory infections were associated with long term exposure to outdoor fine particulate matter^33^. In this study, the only region that did not demonstrate a significant association between PM_2.5_ and burden of LRIs was the Eastern Mediterranean region. Khader stated that, although air pollution such as PM_2.5_ is recognized to be a global health problem, it is difficult to find evidence of its health effects due to the lack of data on air pollution in that region^64^. There are several reasons, including that the impact of air pollution on health is not perceived there to be a priority for health studies, as well as that there are shortcomings in assessing exposure-outcomes in that region. Because the Eastern Mediterranean region has different characteristics from the rest of the world not only in regard to policies for health data but also in regard to geographical conditions, this affects any exposure assessments of the region. Hence, we note this as a limitation to be considered in future studies.

Lastly, when assessing levels of greenness exposure globally, we found a positive relationship between PM_2.5_ and LRIs in countries with low levels of greenness as compared to countries with high levels of greenness. When focusing on analysis by region, we found significant positive associations in African, American, European, and Western Pacific countries with low levels of greenness exposure. Numerous studies have explained the protective effects of greenness exposure in decreasing PM_2.5_ concentrations^65,66^ and in reducing general health burdens and specific health burdens, such as respiratory diseases^28,67^. A cohort study conducted in China researched the interaction between residential greenness and mortality related to air pollution^68^. The study noted the synergistic effect of greenness and concluded that controlling air pollution helps to improve the public’s health and well-being^68^. Nonetheless, there were no significant associations in the Southeast Asian or Eastern Mediterranean regions regardless of greenness levels. For Southeast Asia, we suspected a significant association would not be found because the sample size was too small to conduct a stratification analysis. Faber and Fonesca previously described the phenomenon that small sample size can undermine the internal and external validity of a study and, thus, reduce statistical power^69^.

Several limitations should be noted. First, a country-level database may not be the most prudent area-level selection for assessments of the study variables; in the case of disease burden due to LRIs, a database is provided for the world at the city, county, and township level, so it may be wiser to consider a more granular level than the country-level in future studies. Second, this study lacks data from direct observation; and, for purposes of model development, especially for regional analyses, observational data of PM_2.5_ from monitoring stations is preferred because of its accuracy. Third, since this ecological study used estimates at the country-level, health data at the individual level was lacking, and that may have impacted the strength of the evidence. Fourth, several confounding variables that affect lower respiratory infections have not been considered, including genetics, race/ethnicity, HIV status, etc. It is suggested future studies include these aforementioned confounding variables. In its spatial pattern and hotspot analyses, this study could only solve territory variation issues in terms of polygon size, and it was not able to deal with discontinuity problems for countries that did not have neighboring borders, such as Australia, New Zealand, etc. Consideration of an appropriate weight matrix is recommended for future studies. Even though some shortcomings have been presented following our findings, this study can serve as scientific evidence and a contribution to the knowledge base of critical locations of LRIs and to understand how exposure to PM_2.5_ can increase health problems in terms of the burden due to LRIs globally. Finally, this study offers recommendations for increasing exposure to greenness in an effort to reduce PM_2.5_ concentrations, which can sustainably alleviate general health burdens and specific health burdens, such as LRIs.

## Methods

### Lower respiratory infection database

The disability-adjusted life year (DALY) database of 183 countries was obtained for analysis from the World Health Organization (WHO)^70^. The estimated value of DALY due to LRIs was used to represent the burden of LRIs in each country. DALY is a summary metric of population health comprised of two metrics, namely years of life lost due to premature mortality (YLL) and years lived with disability (YLD)^35^. DALY represents an absolute measure of health loss by counting how many years of healthy life have been lost due to non-fatal illness, impairment, and death. The data for DALY was collected at three different levels (national, regional, and global) in four different years (2000, 2010, 2015, and 2016). Data for 183 WHO countries (Supplementary Table S1) within six WHO regions (Africa, America, Eastern Mediterranean, Europe, Southeast Asia, and Western Pacific) were extracted for this study’s analysis of data at the global level (Supplementary Fig. S1). This study targets LRIs because they are a major cause globally of mortality and have a relationship with environmental exposures^71,72^. The International Classification of Diseases 10th revision codes (ICD-10) was used in order to identify LRIs. Pneumonia and Bronchiolitis, serving as the primary LRIs^2^, were identified via codes J09-J22, P23, and U04. The spatial distribution for each time frame of DALY due to LRIs is illustrated in Supplementary Fig. S2.

### Fine particulate matter assessment

To estimate country-level PM_2.5_, we used satellite image data provided by the Atmosphere Composition Analysis Group, which was established by Prof. Randall Martin from Dalhousie University. This data has a spatial resolution of 1-km, which means that one pixel represents an area size on the ground of one-by-one kilometer. To extract the PM_2.5_ concentration for each country from this image, we applied the function ‘zonal statistics as table’ in ArcGIS and used country boundaries as a feature layer defining the zones. As basic information, this available PM_2.5_ data was assessed from satellite images via a Geographically Weighted Regression (GWR) adjustment and was processed using a validated method that combined the daily total column of aerosol optical depth (AOD) retrievals from the National Aeronautics and Space Administration (NASA)—Moderate Resolution Imaging Spectroradiometer (MODIS), Multi-angle Imaging Spectroradiometer (MISR), and Sea-viewing Wide Field-of-view Sensor (SeaWiFS) with the GEOS-Chem chemical transport model^73^. The data produced using this method had the advantage that it was available for all regions, and, thus, was appropriate for our study at the global level. Furthermore, in line with health outcomes being included for each of the four collection periods, information for country-level PM_2.5_ was accessed in 2000, 2010, 2015, and 2016. Supplementary Fig. S3 displays the geographical distribution of PM_2.5_ exposure for each country during each year of data collection.

### Dataset of covariates

Several country-level variables that had been identified as covariates in prior studies were included in this study to investigate the association globally between PM_2.5_ and LRIs. This study included demographic factors that have been demonstrated to be related to health outcomes, such as population density, age, and sex^74,75^, and the data for these were provided by the United Nations. For investigating the effect of sex, we set male as the reference category^76^. Concerning socioeconomic status (SES), previous studies have indicated a strong relationship between SES and prevalence of infectious diseases^5,77^, such as LRIs. Data for income levels obtained from the World Bank Group were used to represent socioeconomic status. Healthcare utilization is another significant factor in health outcomes^78,79^. Therefore, total healthcare expenditure as the share of national Gross Domestic Product (% of GDP) including the provision of health services (preventive and curative), family planning activities, nutrition activities, and emergency aid designated for health which provided by World Bank Group was taken into account. Lifestyle behaviors such as smoking and alcohol consumption have been demonstrated to increase the burden of LRIs^80–82^; accordingly, the prevalence rate of smoking and the average amount of alcohol consumption in liters per population, as provided by the World Bank Group, were controlled for in the model. Lastly, meteorological factors can trap air pollutants and further facilitate the acquisition and negative effects of respiratory diseases^83–86^. Therefore, we incorporated temperature data obtained from the Climatic Research Unit of the University of East Anglia-The Climate Change Knowledge Portal. Moreover, the global wind atlas (GWA 3.0), developed by Badger and his team, was also included to estimate wind speed^87^. It is noted that covariates were accessed during the same study periods so as to be comparable to health outcomes from the same study periods.

### Analysis of spatial pattern and hotspots

Spatial statistical approaches, including spatial pattern and cluster mapping, were performed in our study. These methods are important in epidemiological studies to identify potential locations for the spread of communicable diseases such as lower respiratory infections^15^. These methods are common in investigating the spatial distribution of environmental exposures^88–90^. Using the estimated DALY adjusted for the population, the spatial autocorrelation Global Moran’s I was used to assess the spatial pattern, degree of clustering, and randomness of the LRI burden globally. Moran’s I is generally preferred over Geary’s C because the values of the former are more intuitive in measuring spatial autocorrelation^91^. The value of Moran’s index generally varies between − 1 and 1^92^. Positive autocorrelation occurs when similar values cluster together and a negative autocorrelation occurs when dissimilar values cluster. A value near zero means there is no autocorrelation at all. Moran's I has been found to provide a high level of performance in spatial statistical analysis and can be used to complement traditional geostatistical model^11,12^. This spatial analysis follows Tobler’s (1970) law that “everything is related to everything else, but near things are more related than distant things.” This analysis was computed with a row-standardized spatial weights matrix that was based on critical distance thresholds. Given the importance of borders in the study of global conflict, the data used was potentially biased due to sampling design and an imposed aggregation scheme^93,94^. A fixed distance band was then considered because it is a prudent option for data analysis when there is a large888 variation in polygon size. The following is a Global Moran’s formula,

1 $$I = \frac{{n\sum\nolimits_{i = 1}^{n} {\sum\nolimits_{j = 1}^{n} {\mathop w\nolimits_{i.j} \mathop z\nolimits_{{\mathbf{i}}} \mathop z\nolimits_{j} } } }}{{\mathop s\nolimits_{0} \sum\nolimits_{i = 1}^{n} {\mathop z\nolimits_{i}^{2} } }},$$

where *I* is Moran’s index, *Z*_*i*_ and *Z*_*j*_ are the deviations for country *i* and *j* from the mean (*X*_*i*_–$\stackrel{-}{X}$) and (*X*_*j*_–$\stackrel{-}{X}$); *X*_*i*_ and *X*_*j*_ are the numbers of DALY loss due to LRIs for country *i* and *j*; and $\stackrel{-}{X}$ is average value of DALY for all countries, *W*_*ij*_ is the spatial weight between *i* and *j*, *n* is equal to the total number of studied countries, and *S*_*0*_ is the aggregate of all the spatial weights.

For the hotspot analysis, Getis-Ord Gi* was applied to identify the statistically significant hot spots and cold spots of burden from LRIs for all the countries (183 countries) included from the six regions. The resultant z-scores (Gi*) and p-values designate the countries with either a high or low value for LRIs. The statistical significance of a z-score assigned to each area identifies the presence and intensity of local clusters of hot spots and cold spots of LRIs relative to the hypothesis of spatial randomness. Getis-Ord Gi* functions by looking at each feature within the context of neighboring values for the same feature, meaning the number of neighbors that have the same value for an indicator affects the clustering of hot spots and cold spots. If high values within d of other high values dominate the pattern, then the summation will result in high positive z-score values, and vice versa^95^; practically, this confirms a neighbor effect. In this study, hot spots were defined as the areas with the highest LRIs burden and, spatially, whose neighbors had the same high values. Meanwhile, cold spots were the areas with the lowest LRIs burden and, spatially, whose neighbors had the same low values. The Getis-Ord Gi* index was calculated using formula,

2 $$\mathop G\nolimits_{i}^{*} = \frac{{\sum\limits_{j = 1}^{n} {\mathop w\nolimits_{i.j} \mathop x\nolimits_{j} - \chi \sum\limits_{j = 1}^{n} {\mathop w\nolimits_{i.j} } } }}{{s\frac{{\sqrt {\left[ {n\sum\limits_{j = 1}^{n} {\mathop {\mathop w\nolimits^{{^{2} }} }\nolimits_{i.j} - \left( {\sum\limits_{j = 1}^{n} {\mathop w\nolimits_{i.j} } } \right)^{2} } } \right]} }}{n - 1}}},$$

where *X*_*i*_ and *X*_*j*_ is number of DALY loss due to LRIs for country *i* and *j*, *W*_*ij*_ is the spatial weight between *i* and *j*, and *n* is equal to the total number of areas analyzed. Moran's I can be expressed in terms of the local Gi* values.

### Statistical model and subgroup analysis

Descriptive statistics were presented to summarize the characteristics of the variables examined in this study, and these include data of major exposures and outcomes—PM_2.5_ and DALY loss due to LRIs, demographic factors, socioeconomic, healthcare status, behaviors, and meteorological factors. A generalized linear mixed model (GLMM) with a penalized quasi-likelihood (PQL) was applied in order to generate the study’s model in investigating the association between PM_2.5_ and LRIs. In addition to offering a flexible approach, the GLMM was selected because this algorithm can consider both fixed and random effects in its calculation and has been applied in several environmental exposure studies related to health outcomes^97–99^, as well as has widely been used in air pollution epidemiology studies^100–102^. For model structure, we set DALY loss due to LRIs as the dependent variable (*Y*) and PM_2.5_ as the main predictor (*X*_*1*_). Several fixed effect covariates, such as demographic, socioeconomic, healthcare status, lifestyle behaviors, and meteorological factors, were further examined. The unit of the dependent variable (DALY) is “years”, measured as a continuous variable, and our GLMMPQL model is based on a linear regression model. Country ID serves as the clustering unit and was treated as a random intercept and used to minimize the temporal correlation of outcomes due to repeated measurements within a country. In this case, random effect and residual error were assumed to have a multivariate normal distribution (Gaussian distribution). To model the yearly temporal variance–covariance structure, the continuous-time first-order autoregressive model, denoted AR (1), was used. Furthermore, in the case where spatial data were provided from distinct areas, in most popular implementations, a GLMMPQL can adjust the overall fixed effects while the structure of correlation remains nested within regions and allows for spatial autocorrelation only between observations in the same region^103^, correlation between neighbors can be included in Bayesian implementations of GLMM models. Therefore, to deal with the spatial autocorrelation problem, we also added the term of the ‘continent’ in the GLMMPQL calculation^97^. In addition, to ensure there was no multicollinearity issue across the adjusted covariates, the generalized variance-inflation factors (GVIFs) were then examined. In this study, we obtained GVIFs with a value less than four (< 4) for all covariates^104^. As a result, we included all of those GVIFs values in the Supplementary Table S2.

To investigate the robustness of our association model, we developed six sensitivity models with different covariate settings. In detail, Model 1 only controlled for demographic factors; Model 2 included demographic factors (Model 1) and the proportion of alcohol consumption; Model 3 added the prevalence rate of smoking to Model 2’s inclusions; Model 4 added economic status for each country, as represented by income-level, to Model 3’s considerations; and Model 5 considered healthcare expenditure in addition to Model 4’s criteria. Model 6 adjusted for all covariates but excluded data from Eastern Mediterranean countries. We excluded data from Eastern Mediterranean countries in the sensitivity model to identify whether PM_2.5_ still remains associated with the burden of LRIs after eliminating data from countries with the highest PM_2.5_ (Supplementary Fig. S5); PM_2.5_ is highly present in Eastern Mediterranean countries because the area is naturally covered by desert dust and has low levels of vegetation^105^.

Subgroup analyses were also conducted in this study to determine whether the association between PM_2.5_ and LRIs exists within various subpopulations. Since prior studies have confirmed cases of LRIs vary by age group and, specifically, children younger than 5 years of age experience LRIs at a disproportionately high rate^106,107^, we conducted stratified tests for five age groups (< 5; 5–14; 15–49; 50–69, and ≥ 70 years). We also performed subgroup analyses for the six WHO regions, which include the African, American, European, Eastern Mediterranean, Southeast Asian, and Western Pacific regions. In addition, subgroup analyses were performed across regions comparing the relationship between PM_2.5_ and LRIs in areas with low and high levels of greenness. The greenness effect was considered in this stratified analysis because prior studies have stated that greenness can reduce the concentration of air pollutants such as PM_2.5_ and can directly-indirectly reduce the health burden resulting from lower respiratory infections. For the country-level greenness measurement, we used NDVI data from the Terra Moderate Resolution Imaging Spectroradiometer provided by the National Aeronautics and Space Administration with spatial resolution 1 × 1 km^2^^96^. NDVI images with the acquisition date closer to the mid-season were selected from January, April, July, and October; the selection of the months of data collection was considered for countries with two and/or four seasons. Then, we used the median of NDVI to classify the region as having a low or high exposure to greenness. Furthermore, the median values of NDVI globally and in the various regions are displayed in Supplementary Table S3. The spatial-statistical analyses were completed using ArcGIS 10.7.1 (Esri Inc., 23 Redlands, California, US) and R v. 3.6.3 developed by R Core Team^108^. Coefficient estimates were performed with 95% CI and p-values < 0.05 were deemed to be statistically significant.

## Supplementary Information

Supplementary Information.
