# Supplementary material for: Spatial patterns of lower respiratory tract infections and their association with fine particulate matter
Source: Sci Rep. 2021 Mar 1;11:4866. doi: 10.1038/s41598-021-84435-y (PMC7921673; doi:10.1038/s41598-021-84435-y)
Supplement: Supplementary file 1 — Supplementary Information. [file 41598_2021_84435_MOESM1_ESM.pdf]

Spatial Patterns of Lower Respiratory Tract Infections and Their Association with Fine Particulate Matter

Aji Kusumaning Asri[1], Wen-Chi Pan[2], Hsiao-Yun Lee[3], Huey-Jen Su[4], Chih-Da Wu[1,5,*], John D. Spengler[6]

[1]National Cheng Kung University, Department of Geomatics, Tainan, 70101, Taiwan

[2]National Yang Ming University, Institute of Environmental and Occupational Health Sciences, Taipei, 11221, Taiwan

[3]National Taipei University of Nursing and Health Sciences, Department of Leisure Industry and Health Promotion, Taipei 112, Taiwan

[4]National Cheng Kung University, Department of Environmental and Occupational Health, Tainan, 70101, Taiwan

[5]National Health Research Institutes, National Institute of Environmental Health Sciences, Miaoli, 35053, Taiwan

[6]Harvard T.H. Chan School of Public Health, Department of Environmental Health, Boston, 02115, USA

[*]chidawu@mail.ncku.edu.tw

Supplementary Information

Table of Contents

Supplementary Table S1. WHO member countries (183 countries).

Supplementary Table S2. Multicollinearity test analysis related to adjusted covariates and lower respiratory infection.

Supplementary Table S3. The median values of NDVI in the various regions and global.

Supplementary Figure S1. The geographical distribution of WHO regions.

Supplementary Figure S2. The geographical distribution of DALY due to lower respiratory infection in 2000 – 2016.

Supplementary Figure S3. The geographical distribution of $PM_{2.5}$ exposure in 2000 – 2016

Supplementary Figure S4. Temporal trends of DALY due to lower respiratory infection by region.

Supplementary Figure S5. Temporal trends of $PM_{2.5}$ exposure by region.

Supplementary Figure S6. Spatial hot spots and cold spots of DALY due to LRI in **(a)** 2000, **(b)** 2010, **(c)** 2015, and **(d)** 2016.

Supplementary Figure S7. Getis-Ord statistics for DALY due to LRI in **(a)** All periods, **(b)** 2000, **(c)** 2010, **(d)** 2015, **(e)** 2016.

Supplementary Table S1. WHO member countries (183 countries).

WHO Regions	Countries
Africa (46 countries)	Algeria, Angola, Benin, Botswana, Burkina Faso, Burundi, Cameroon, Central Africa, Chad, Comoros, Congo, Cote d'lvoire, D. Republic Congo, Eq. Guinea, Eritrea, Ethiopia, Gabon, Gambia, Ghana, Guinea, Guinea-Bissau, Kenya, Lesotho, Liberia, Madagascar, Malawi, Mali, Mauritania, Mauritius, Mozambique, Namibia, Niger, Nigeria, Rwanda, Sao Tome and Principe, Senegal, Seychelles, Sierra Leone, South Africa, South Sudan, Swaziland, Togo, Uganda, United Rep. Tanzania, Zambia, Zimbabwe.
America (34 countries)	Antigua and Barbuda, Argentina, Bahamas, Barbados, Belize, Bolivia, Brazil, Costa Rica, Canada, Cape Verde, Chile, Colombia, Cuba, Dominican Rep., Ecuador, El Salvador, Grenada, Guatemala, Guyana, Haiti, Honduras, Jamaica, Mexico, Nicaragua, Panama, Paraguay, Peru, Saint Lucia, Saint Vincent and the Grenadines, Suriname, Trinidad and Tobago, USA, Uruguay, Venezuela.
South-East Asia (11 countries)	Bangladesh, Bhutan, Democratic Rep. Korea, India, Indonesia, Maldives, Myanmar, Nepal, Sri Lanka, Thailand, Timor-Leste.
Europe (50 countries)	Albania, Armenia, Austria, Azerbaijan, Belarus, Belgium, Bosnia, Bulgaria, Croatia, Cyprus, Czechia, Denmark, Estonia, Finland, France, Georgia, Germany, Greece, Hungary, Iceland, Ireland, Israel, Italy, Kazakhstan, Kyrgyzstan, Latvia, Lithuania, Luxembourg, Malta, Montenegro, Netherlands, Norway, Poland, Portugal, Rep. Moldova, Romania, Russian Fed., Serbia, Slovakia, Slovenia, Spain, Sweden, Switzerland, Tajikistan, Yugoslav, Turkey, Turkmenistan, Ukraine, UK, Uzbekistan.
Eastern Mediterranean (21 countries)	Afghanistan, Bahrain, Djibouti, Egypt, Iran, Iraq, Jordan, Kuwait, Lebanon, Libya, Morocco, Oman, Pakistan, Qatar, Saudi Arabia, Somalia, Sudan, Syrian Arab Rep., Tunisia, United Arab Emirates, Yemen
Western Pacific (21 countries)	Australia, Brunei Darussalam, Cambodia, China, Fiji, Japan, Kiribati, Lao, Malaysia, Micronesia, Mongolia, New Zealand, Papua New Guinea, Philippines, Rep. Korea, Samoa, Singapore, Solomon, Tonga, Vanuatu, Vietnam.

Supplementary Table S2. Multicollinearity test analysis related to adjusted covariates and lower respiratory infection.

Variables	GVIFs
**Covariates**	-
Population density (people/km^2)	1.05
Gender (male %)	1.33
Age 0 - 4 (years, %)	2.52
Age 5 - 14 (years, %)	3.58
Age 15 - 49 (years, %)	2.62
Age 50 - 69 (years, %)	1.92
Age > 70 (years, %)	2.25
Economic – income level	1.15
Alcohol consumption (liters/population/year)	1.15
Smoking (%)	1.14
Continent	1.20
Temperature (ºC)	1.32
Wind speed (m/s)	1.10
Healthcare expenditure (% of GDP)	1.13
**Main exposures**	-
$PM_{2.5}$ (µg/m^3)	1.24

Supplementary Table S3. The median values of NDVI in the various regions and global.

	Median of NDVI
**Global**	**0.545**
Low exposure to greenness	< 0.545
High exposure to greenness	≥ 0.545
**Africa**	**0.551**
Low exposure to greenness	< 0.551
High exposure to greenness	≥ 0.551
**America**	**0.680**
Low exposure to greenness	< 0.680
High exposure to greenness	≥ 0.680
**Eastern Mediterranean**	**0.128**
Low exposure to greenness	< 0.128
High exposure to greenness	≥ 0.128
**Europe**	**0.522**
Low exposure to greenness	< 0.522
High exposure to greenness	≥ 0.522
**Southeast Asia**	**0.602**
Low exposure to greenness	< 0.602
High exposure to greenness	≥ 0.602
**Western Pacific**	**0.728**
Low exposure to greenness	< 0.728
High exposure to greenness	≥ 0.728

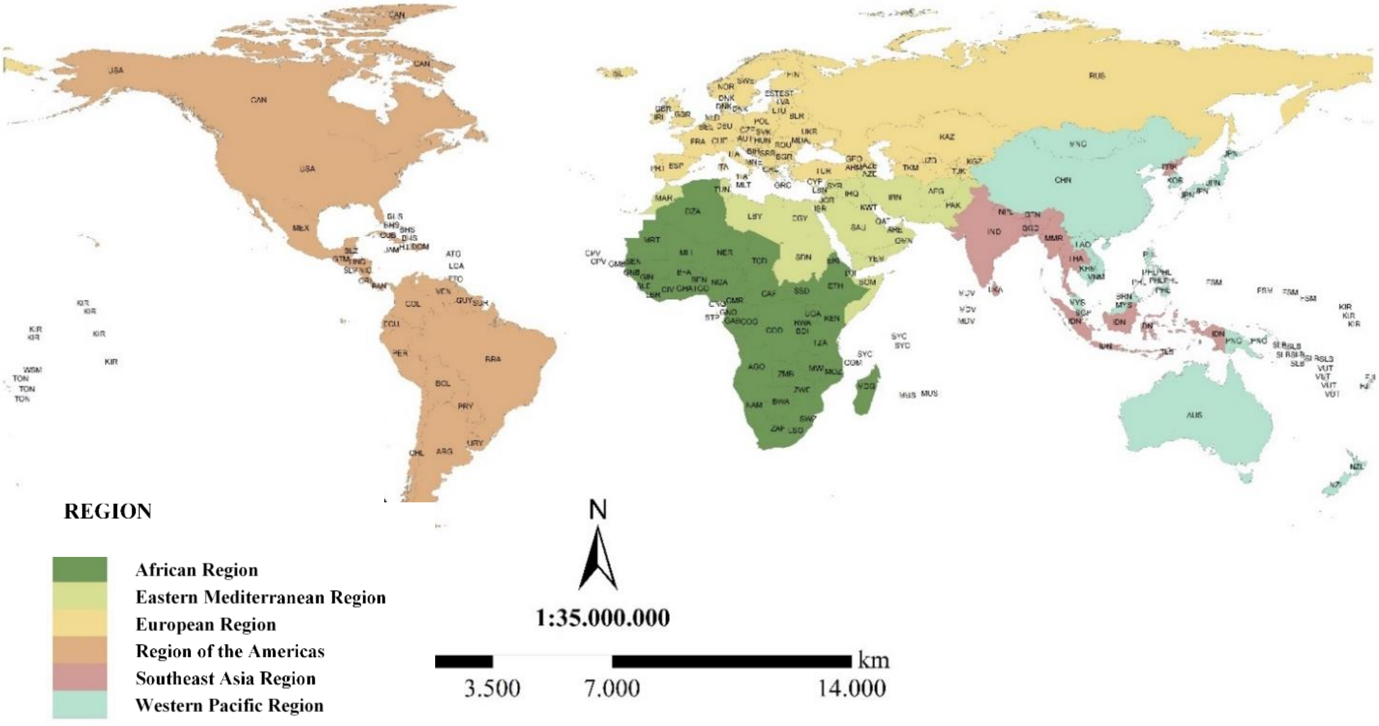

Supplementary Figure S1. The geographical distribution of WHO regions.

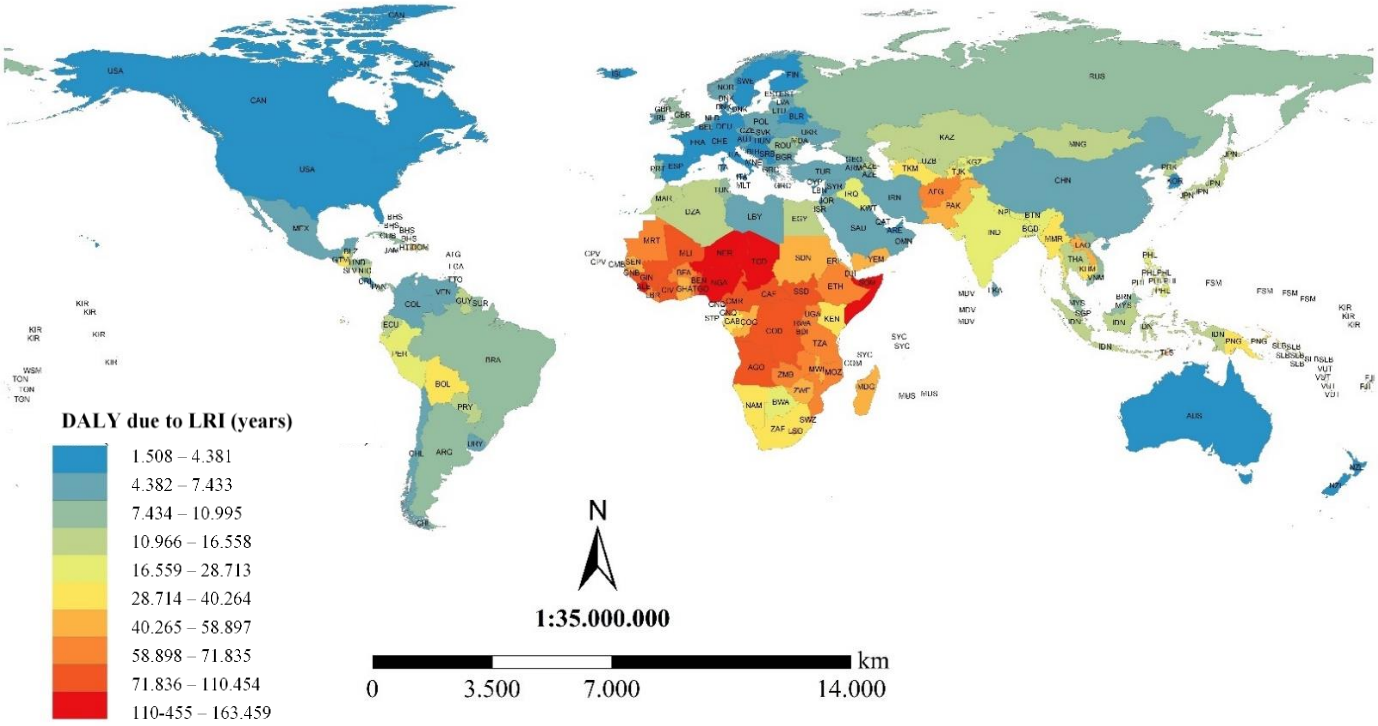

Supplementary Figure S2. The geographical distribution of DALY due to lower respiratory infection in 2000 – 2016.

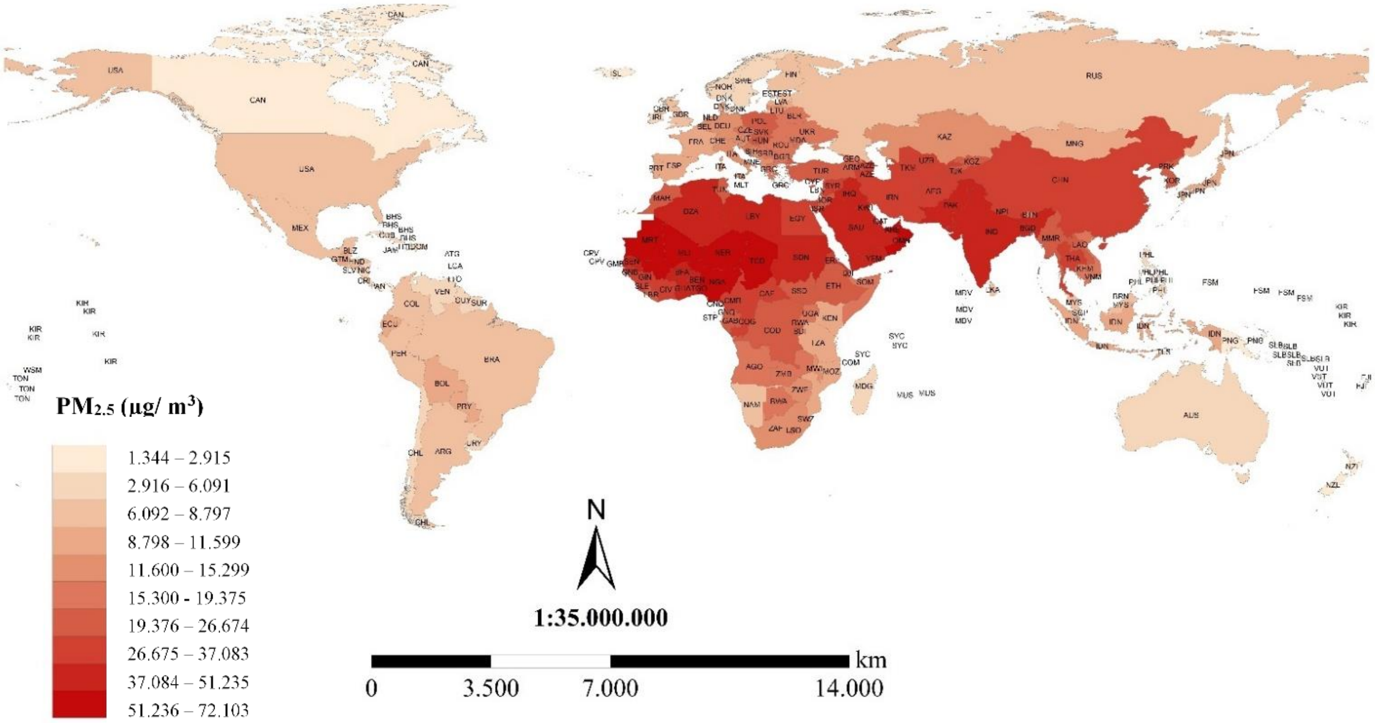

Supplementary Figure S3. The geographical distribution of $PM_{2.5}$ exposure in 2000 – 2016.

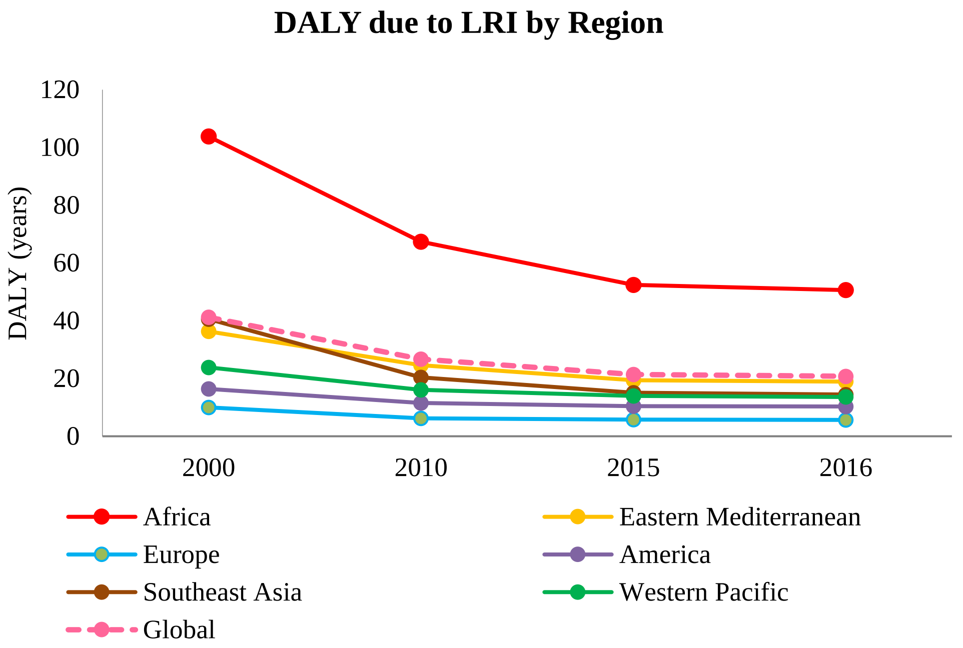

Supplementary Figure S4. Temporal trends of DALY due to lower respiratory infection by region.

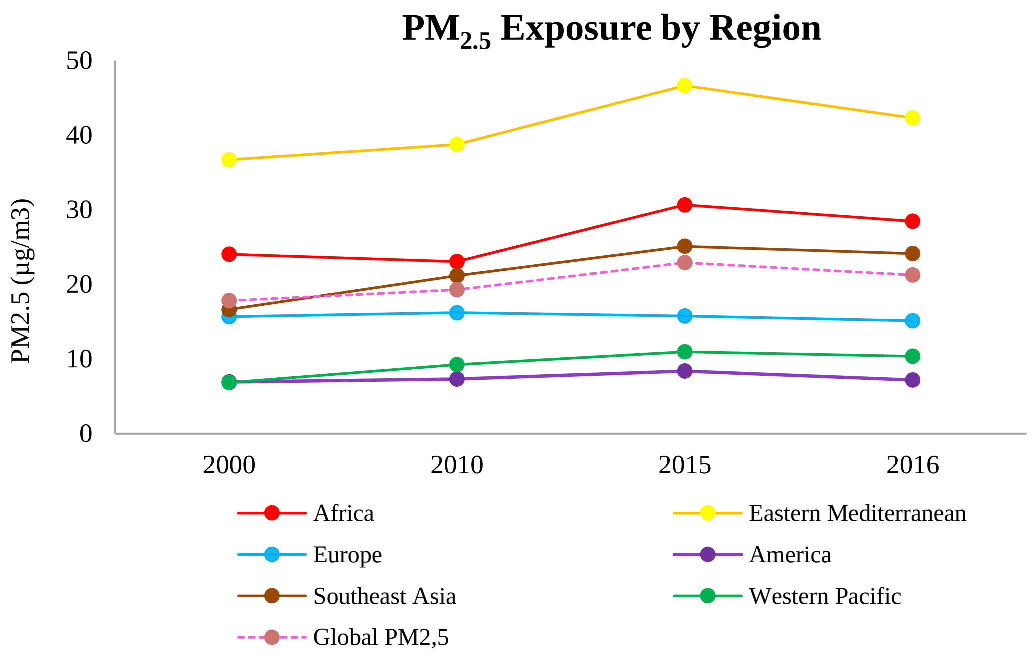

Supplementary Figure S5. Temporal trends of $PM_{2.5}$ exposure by region.

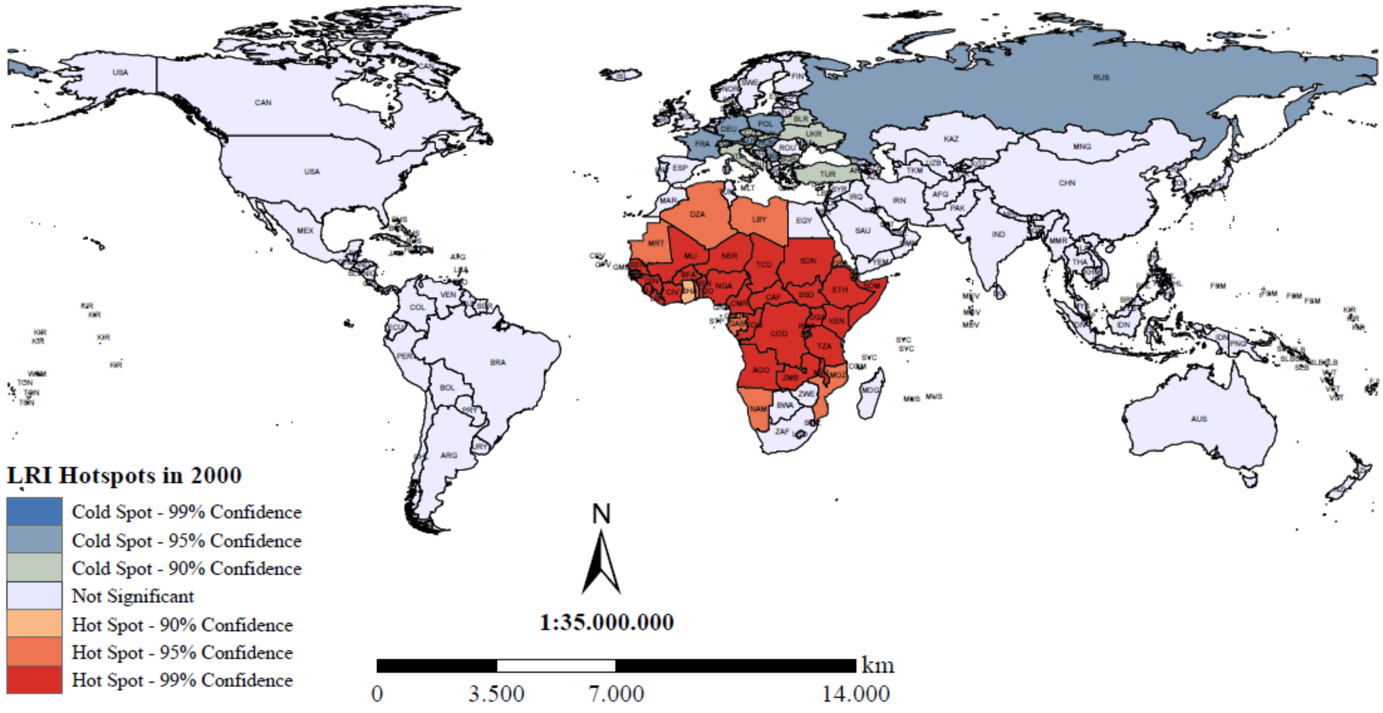

(a)

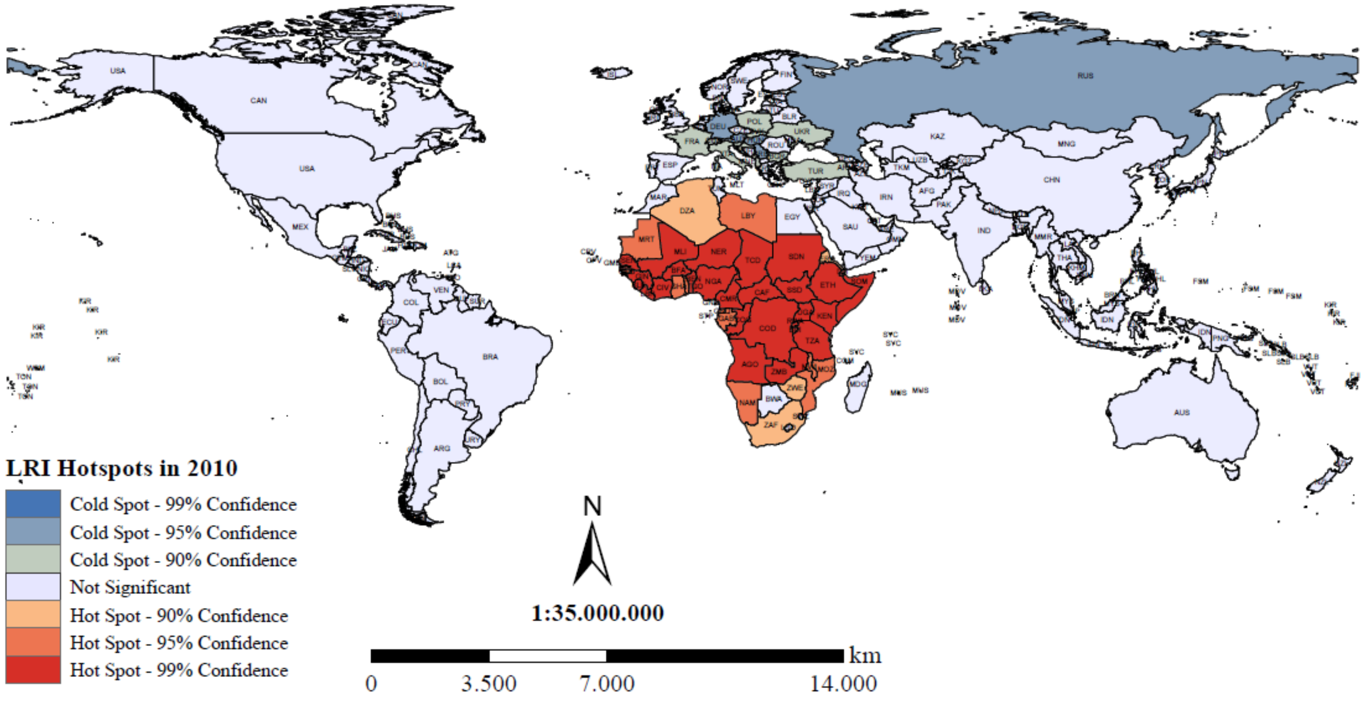
LRI Hotspots in 2010
Cold Spot - 99% Confidence
Cold Spot - 95% Confidence
Cold Spot - 90% Confidence
Not Significant
Hot Spot - 90% Confidence
Hot Spot - 95% Confidence
Hot Spot - 99% Confidence
N
1:35.000.000
km
0
3.500
7.000
14.000

(b)

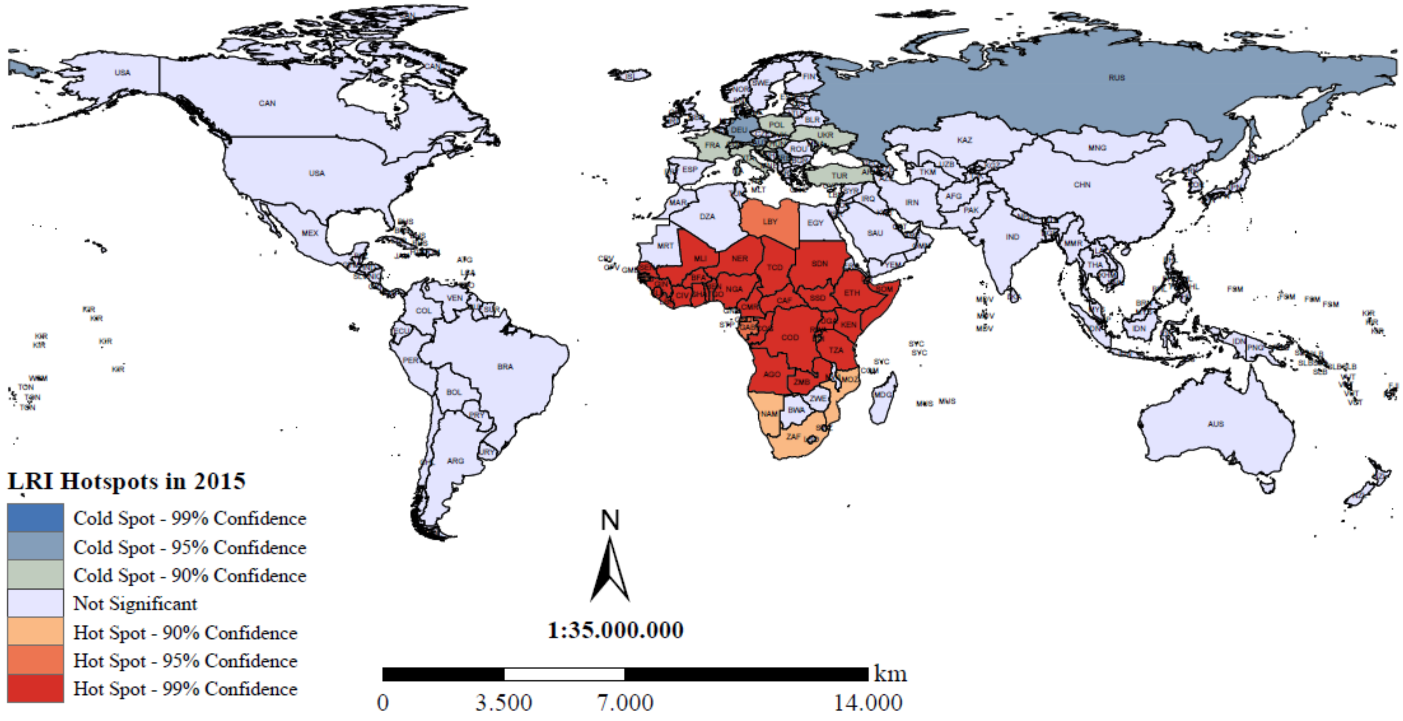
LRI Hotspots in 2015
Cold Spot - 99% Confidence
Cold Spot - 95% Confidence
Cold Spot - 90% Confidence
Not Significant
Hot Spot - 90% Confidence
Hot Spot - 95% Confidence
Hot Spot - 99% Confidence
N
1:35.000.000
km
0
3.500
7.000
14.000

(c)

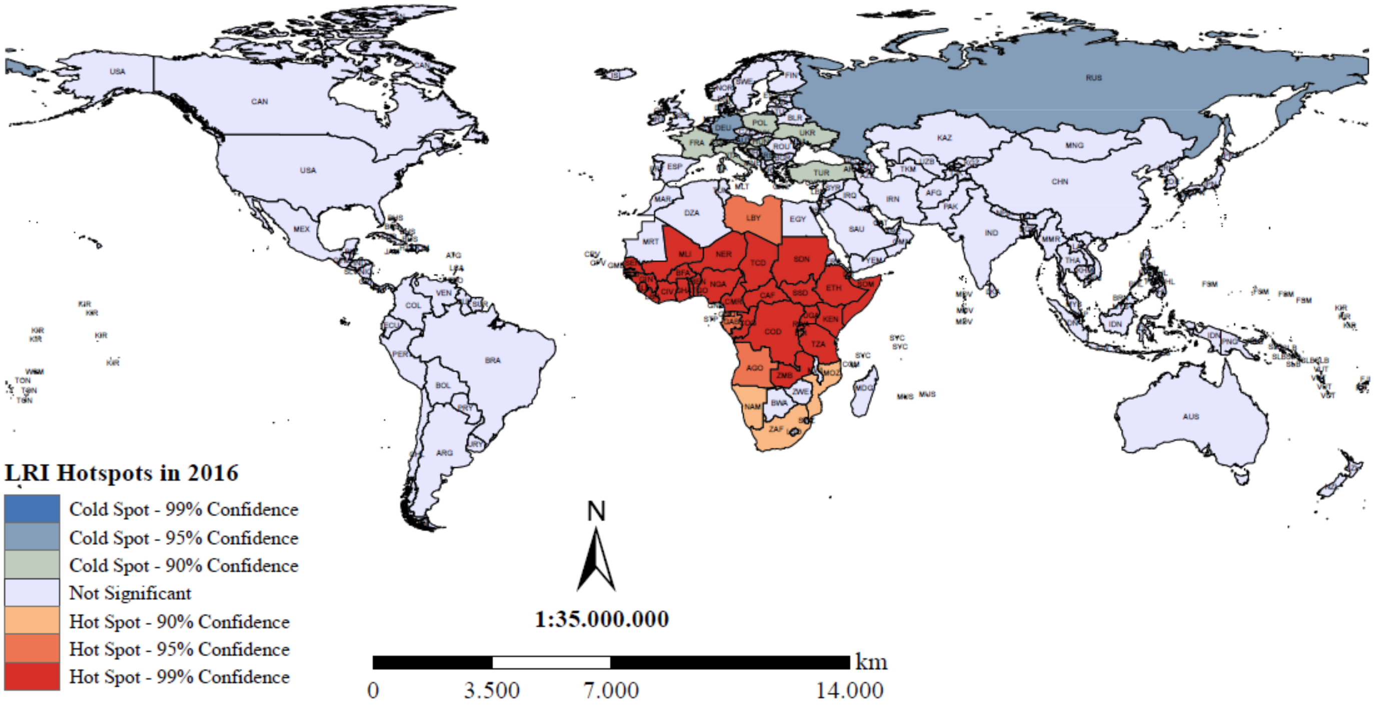

(d)

Supplementary Figure S6. Spatial hot spots and cold spots of DALY due to LRI in **(a)** 2000, **(b)** 2010, **(c)** 2015, and **(d)** 2016.

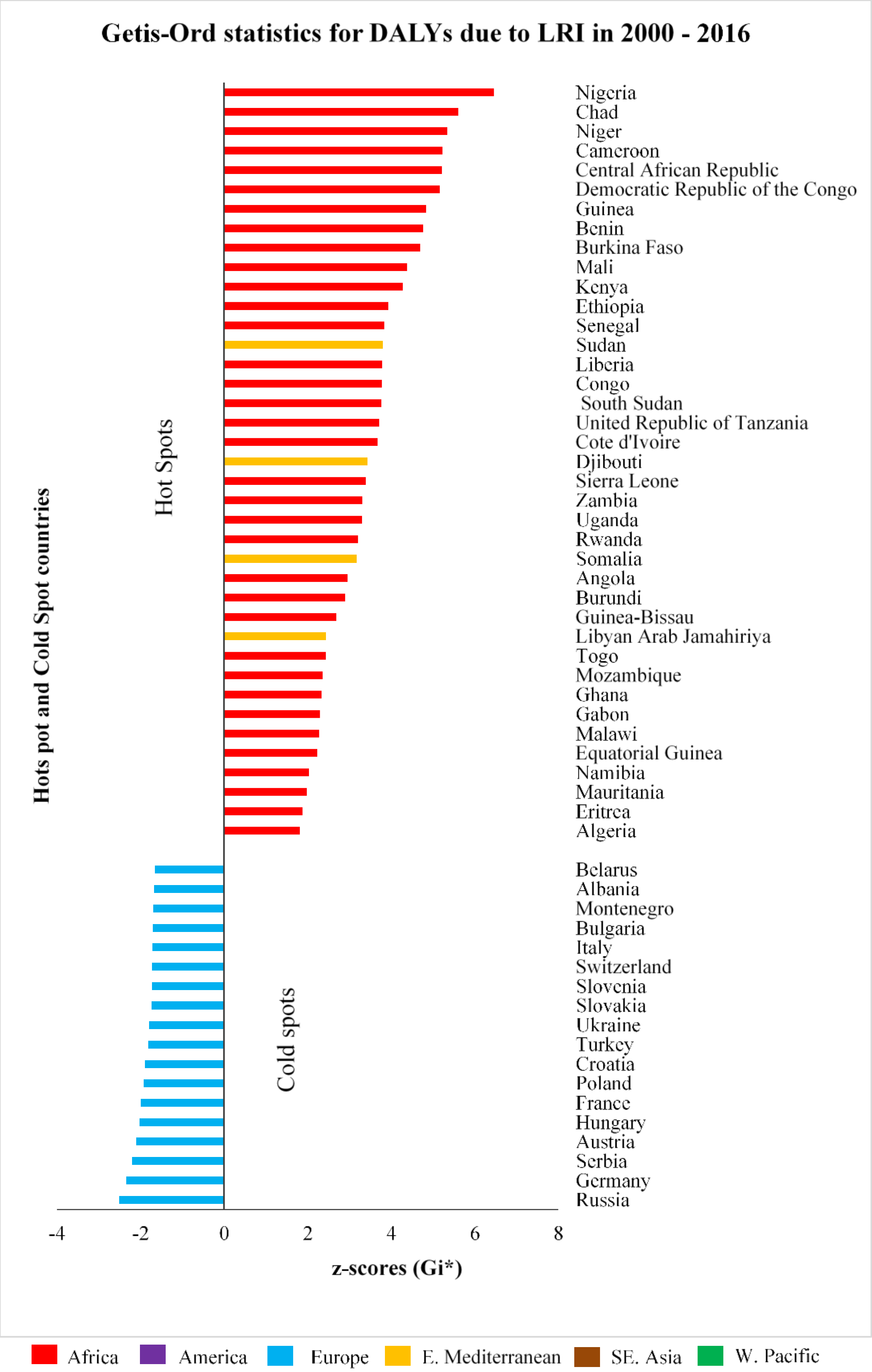
Getis-Ord statistics for DALYs due to LRI in 2000 - 2016
Hots pot and Cold Spot countries
Hot Spots
Cold spots
Nigeria
Chad
Niger
Cameroon
Central African Republic
Democratic Republic of the Congo
Guinea
Benin
Burkina Faso
Mali
Kenya
Ethiopia
Senegal
Sudan
Liberia
Congo
South Sudan
United Republic of Tanzania
Cote d'Ivoire
Djibouti
Sierra Leone
Zambia
Uganda
Rwanda
Somalia
Angola
Burundi
Guinea-Bissau
Libyan Arab Jamahiriya
Togo
Mozambique
Ghana
Gabon
Malawi
Equatorial Guinea
Namibia
Mauritania
Eritrea
Algeria
Belarus
Albania
Montenegro
Bulgaria
Italy
Switzerland
Slovenia
Slovakia
Ukraine
Turkey
Croatia
Poland
France
Hungary
Austria
Serbia
Germany
Russia
-4
-2
0
2
4
6
8
z-scores (Gi*)
Africa
America
Europe
E. Mediterranean
SE. Asia
W. Pacific

(a)

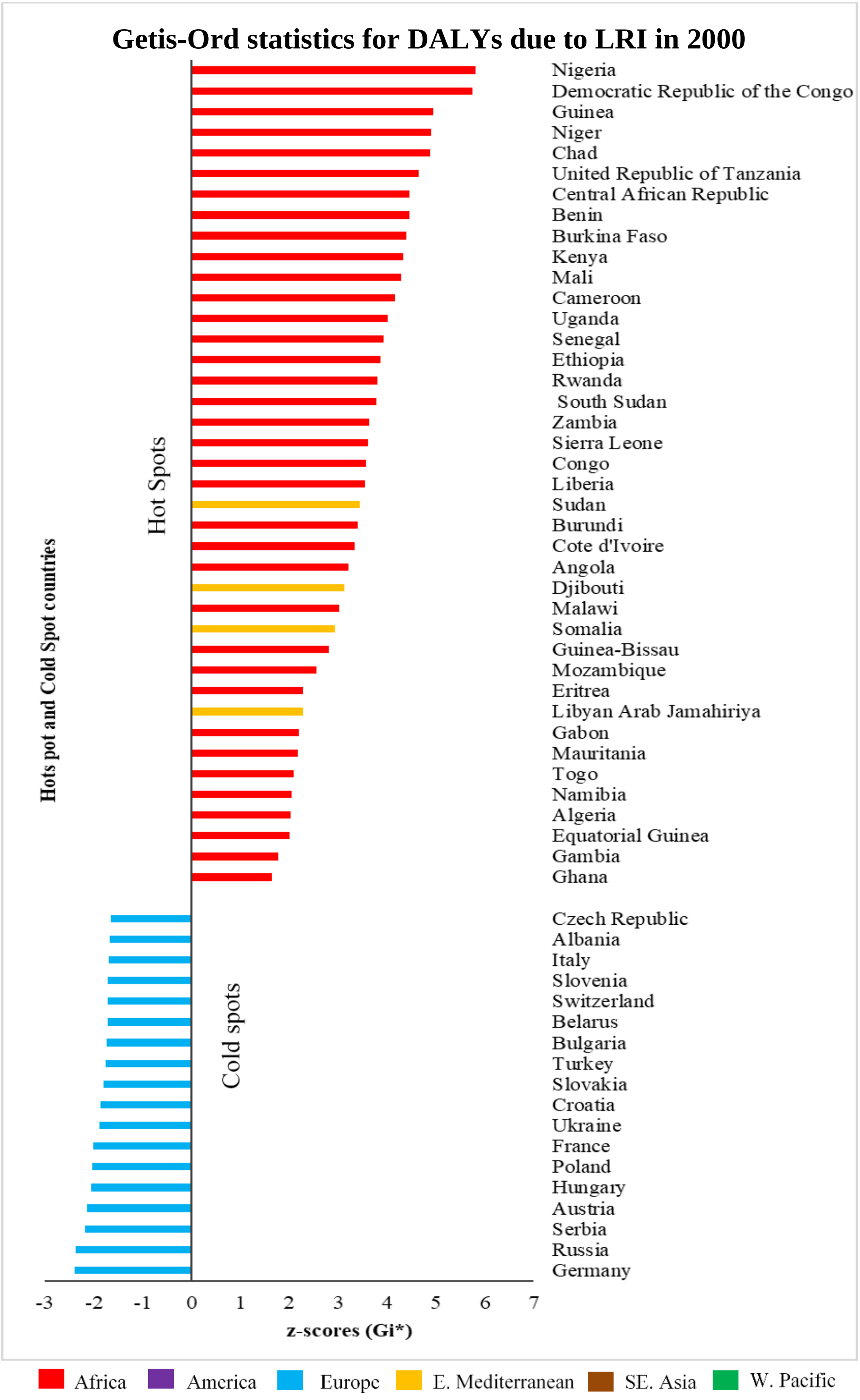

Getis-Ord statistics for DALYs due to LRI in 2000
Nigeria
Democratic Republic of the Congo
Guinea
Niger
Chad
United Republic of Tanzania
Central African Republic
Benin
Burkina Faso
Kenya
Mali
Cameroon
Uganda
Senegal
Ethiopia
Rwanda
South Sudan
Zambia
Sierra Leone
Congo
Liberia
Sudan
Burundi
Cote d'Ivoire
Angola
Djibouti
Malawi
Somalia
Guinea-Bissau
Mozambique
Eritrea
Libyan Arab Jamahiriya
Gabon
Mauritania
Togo
Namibia
Algeria
Equatorial Guinea
Gambia
Ghana
Czech Republic
Albania
Italy
Slovenia
Switzerland
Belarus
Bulgaria
Turkey
Slovakia
Croatia
Ukraine
France
Poland
Hungary
Austria
Serbia
Russia
Germany
Hot Spots
Cold spots
Hots pot and Cold Spot countries
-3 -2 -1 0 1 2 3 4 5 6 7
z-scores (Gi*)
Africa
America
Europe
E. Mediterranean
SE. Asia
W. Pacific

(b)

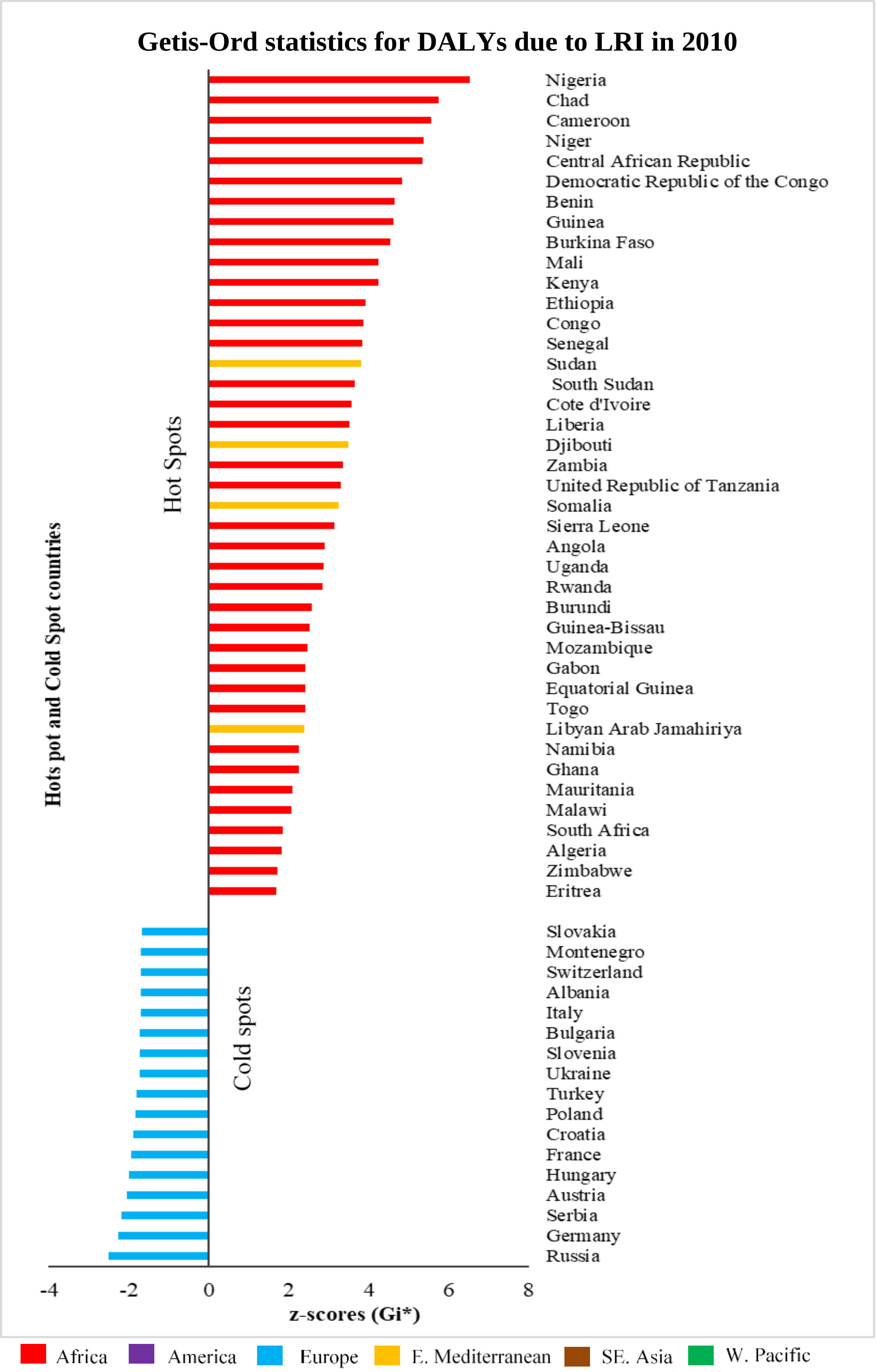
Getis-Ord statistics for DALYs due to LRI in 2010
Hots pot and Cold Spot countries
Hot Spots
Cold spots
Nigeria
Chad
Cameroon
Niger
Central African Republic
Democratic Republic of the Congo
Benin
Guinea
Burkina Faso
Mali
Kenya
Ethiopia
Congo
Senegal
Sudan
South Sudan
Cote d'Ivoire
Liberia
Djibouti
Zambia
United Republic of Tanzania
Somalia
Sierra Leone
Angola
Uganda
Rwanda
Burundi
Guinea-Bissau
Mozambique
Gabon
Equatorial Guinea
Togo
Libyan Arab Jamahiriya
Namibia
Ghana
Mauritania
Malawi
South Africa
Algeria
Zimbabwe
Eritrea
Slovakia
Montenegro
Switzerland
Albania
Italy
Bulgaria
Slovenia
Ukraine
Turkey
Poland
Croatia
France
Hungary
Austria
Serbia
Germany
Russia
-4
-2
0
2
4
6
8
z-scores (Gi*)
Africa
America
Europe
E. Mediterranean
SE. Asia
W. Pacific

(c)

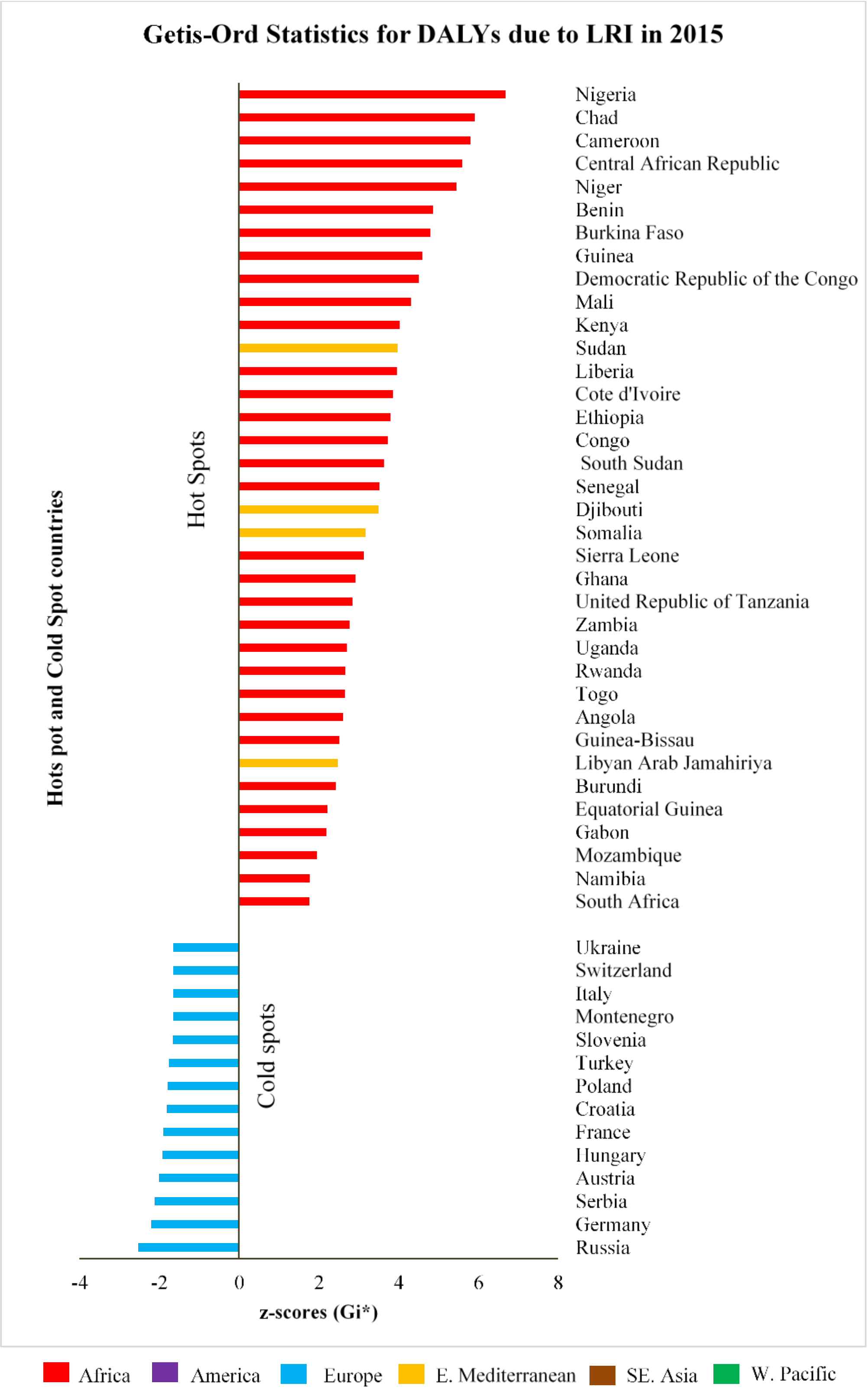

Getis-Ord Statistics for DALYs due to LRI in 2015
Hots pot and Cold Spot countries
Hot Spots
Cold spots
Nigeria
Chad
Cameroon
Central African Republic
Niger
Benin
Burkina Faso
Guinea
Democratic Republic of the Congo
Mali
Kenya
Sudan
Liberia
Cote d'Ivoire
Ethiopia
Congo
South Sudan
Senegal
Djibouti
Somalia
Sierra Leone
Ghana
United Republic of Tanzania
Zambia
Uganda
Rwanda
Togo
Angola
Guinea-Bissau
Libyan Arab Jamahiriya
Burundi
Equatorial Guinea
Gabon
Mozambique
Namibia
South Africa
Ukraine
Switzerland
Italy
Montenegro
Slovenia
Turkey
Poland
Croatia
France
Hungary
Austria
Serbia
Germany
Russia
-4
-2
0
2
4
6
8
z-scores (Gi*)
Africa
America
Europe
E. Mediterranean
SE. Asia
W. Pacific

(d)

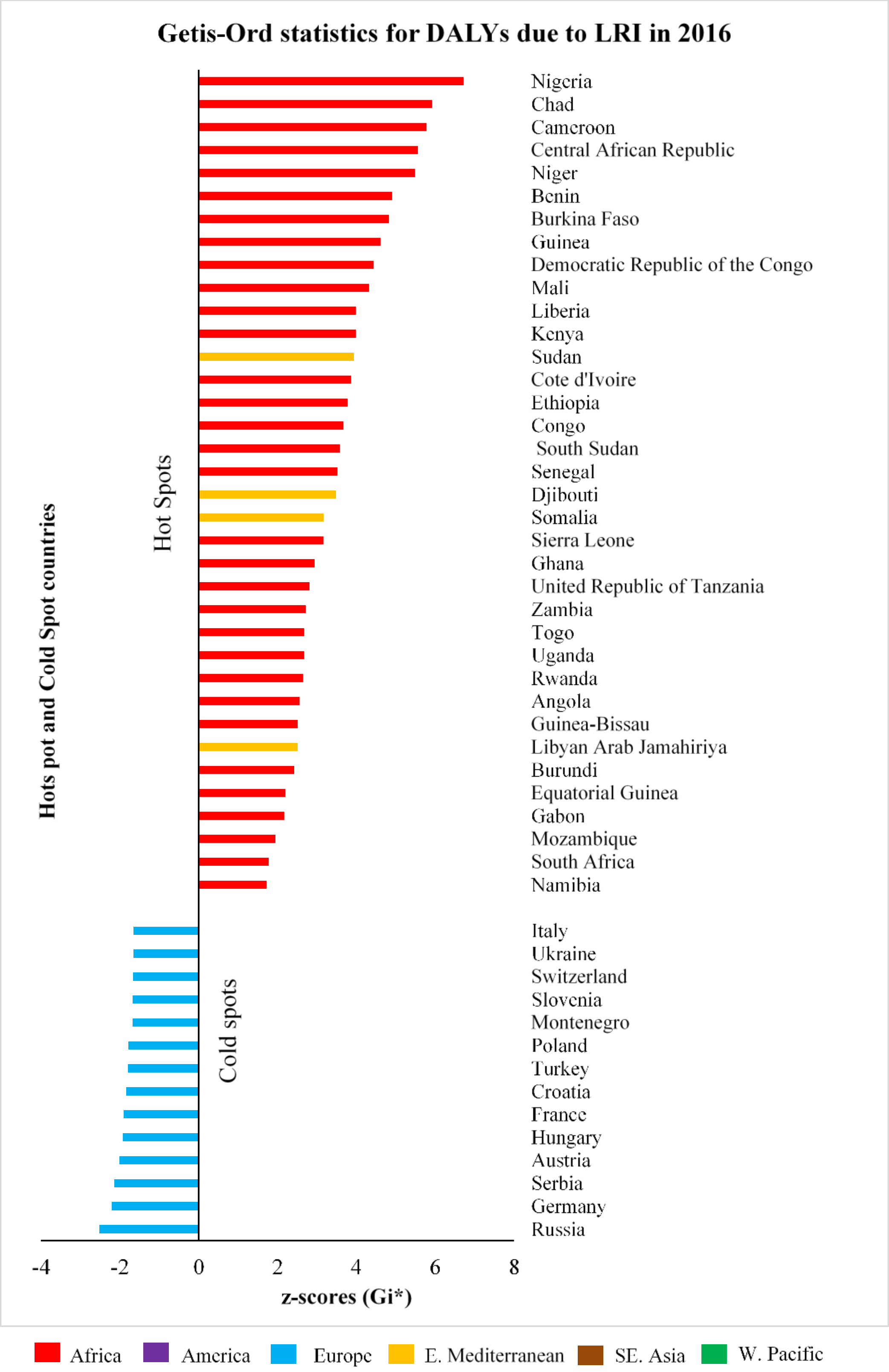

(e)

Supplementary Figure S7. Getis-Ord statistics for DALY due to LRI in **(a)** All periods, **(b)** 2000, **(c)** 2010, **(d)** 2015, **(e)** 2016.
